# Trans-oral trans-sheath forceps biopsy for patients with severe esophageal obstruction under fluoroscopy

**DOI:** 10.1038/s41598-022-22120-4

**Published:** 2022-10-14

**Authors:** Dechao Jiao, Kaihao Xu, Yiming Liu, Zongming Li, Yanli Wang, Jianzhuang Ren, Xinwei Han

**Affiliations:** Department of Interventional Radiology, First Affiliated Hospital of Zhengzhou University, No. 1 Jianshe East Road, Zhengzhou City, 450000 Henan Province China

**Keywords:** Oesophageal cancer, Cancer

## Abstract

To investigate the safety and effectiveness of trans-oral trans-sheath forceps biopsy (TTFB) for patients with severe esophageal obstruction under fluoroscopy. From November 2016 to November 2019, 35 patients with level III or IV dysphagia and a Karnofsky score of less than 60 were enrolled to undergo TTFB and esophageal nutrition tube insertion or stenting simultaneously. Data on diagnostic performance, early complications, and radiation dose were collected, and Karnofsky scores before and after the procedures were compared. The technical success of TTFB was 100%. The sensitivity, specificity and accuracy were 92.3% (24/26), 77.8% (7/9), and 88.6% (31/35), respectively. Complications occurred in two cases (5.7%). The mean procedure duration and irradiation dose were 23.2 min and 7.2 mSv, respectively. The Karnofsky scores significantly increased after 2–4 weeks (t = 11.22, *P* < 0.0001). TTFB is a safe and effective method for patients with severe esophageal obstruction under fluoroscopy, especially in those who cannot undergo or refuse endoscopy.

## Introduction

Esophageal cancer is a common malignant tumor with a poor prognosis; China accounts for more than 50% of esophageal cancers worldwide^1, 2^. The early symptoms of esophageal cancer are not obvious, and most patients are at the middle or late stage when diagnosed. Endoscopy can afford visualization of the esophageal surface, the extent of tumor involvement, ulcer, and diverticulum, and can with the help of intracavitary ultrasound or fluorescence technology facilitate the gathering of samples for a clear diagnosis^3, 4^. Although endoscopic biopsy (EB) has been widely used to obtain samples from the stenosed part, it is not feasible in some patients for various reasons such as patients’ refusal of endoscopic treatment, inability to tolerate anesthesia, and poor constitution, and presence of severe stricture, especially in patients with level 3 or 4 dysphagia and a Karnofsky score less than 60 points. In this study, we introduced a trans-oral trans-sheath forceps biopsy (TTFB) method to obtain esophageal tissue from the occlusion site under local anesthesia.

## Methods

### Patients

All procedures performed in the studies involving human participants were in accordance with the ethical standards of the institutional and/or national research committee and with the 1964 Helsinki Declaration and its later amendments or comparable ethical standards. From November 2016 to November 2019, 35 patients (20 men and 15 women, mean age: 74.3 ± 5.6 years) with level 3 or 4 dysphagia and a Karnofsky score less than 60 were enrolled to undergo TTFB and nutrition tube insertion simultaneously. The reasons for choosing TTFB were poor condition (n = 26), refusal to undergo endoscopy (n = 7), and inability to tolerate anesthesia (n = 2). The mean stenosis length and maximum tumor diameter were 48.7 and 22.3 mm, respectively. More study details are listed in Table 1.

**Table 1:** Study data.

Date	Value (range or %)
Total patients	35
Mean age, years (range)	74.3 ± 5.6 (63–85)
Sex (male/female)	20/15
Location (upper/middle/lower)	9/18/8
Stenosis length (mm)	48.7 ± 14.5 (21–80)
Maximum tumor diameter (mm)	22.3 ± 5.4 (13–37)
Stooler score (III/IV)	16/19
**Previous treatment (%)**	
No	19 (28.6)
Stenting	8 (22.9)
Irradiation	7 (20)
Chemotherapy	4 (11.4)
**The reasons for forceps biopsies**	
Poor conditions	26 (74.2)
Refusal of endoscopy	7 (20)
Anesthesia problems	2 (5.8)
Mean number of biopsies (range)	5.2 (2–8)
Mean procedure duration (min)	21.9 ± 4.5 (15–32)
Mean irradiation dose	7.2 mSv (2.4–11.9)
Balloon dilatation assistance (N/balloon diameter, mm)	7/8
**Karnofsky score**	
Before	51.1 ± 7.6 (40–60)
After	63.1 ± 9.0 (40–80)
Technical success rate (%)	100
Sensitivity (%)	92.3 (24/26)
Specificity (%)	77.8 (7/9)
Accuracy (%)	88.6 (31/35)
Minor bleeding (%)	2
**Final pathology**	
Esophageal carcinoma	28
Granulation tissue	7

### Interventional procedures

**Figure 1:**
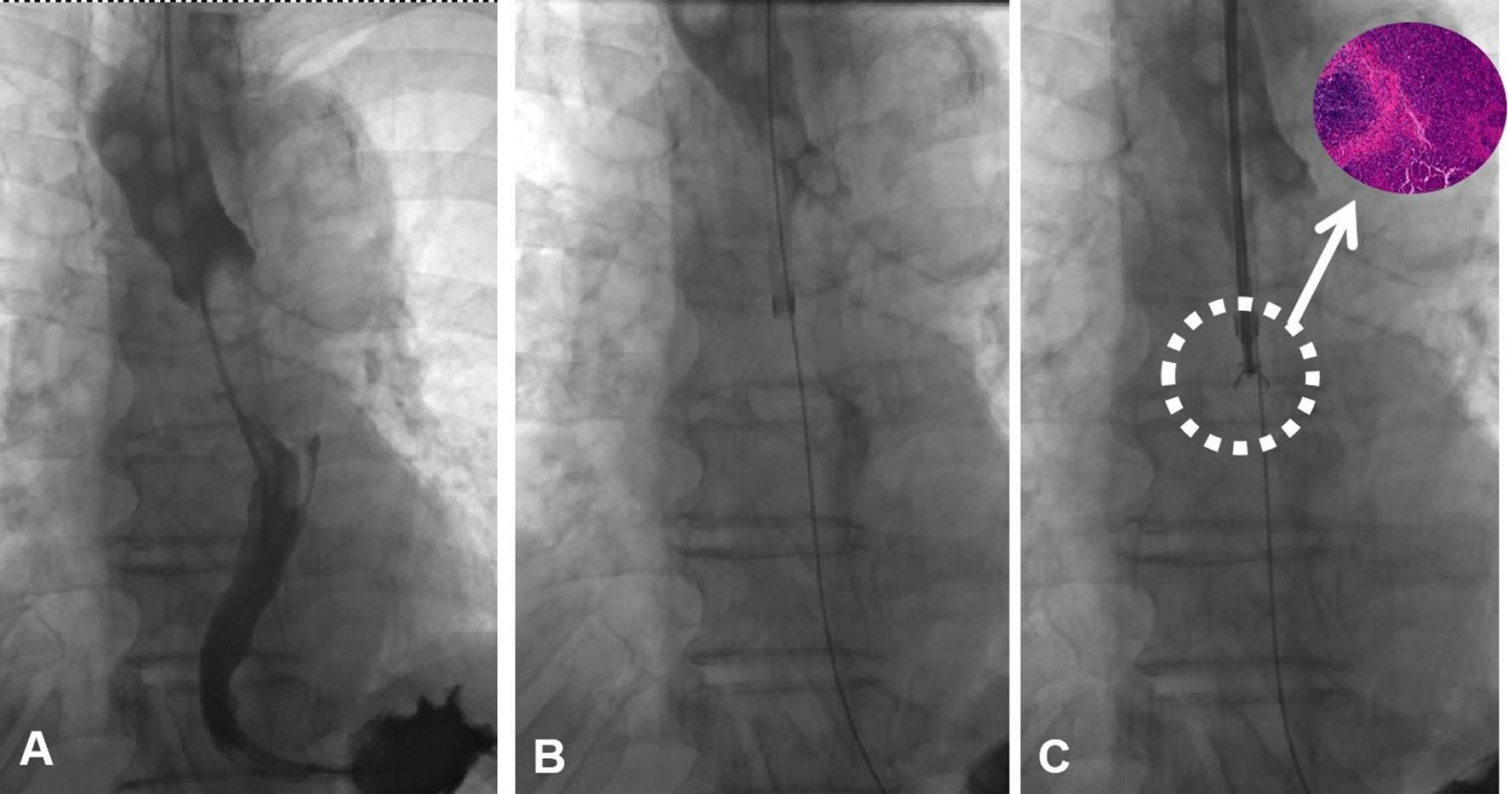
76-year-old male presented with level 3 dysphagia. (**A**) Catheter angiography demonstrated esophageal interruption and filling defect. (**B**) The sheath was introduced along a super-stiff guidewire toward the stenosis part of the lesion. (**C**) The biopsy forceps was introduced through the sheath to clamp the samples, and squamous cell carcinoma was confirmed by pathology.

**Figure 2:**
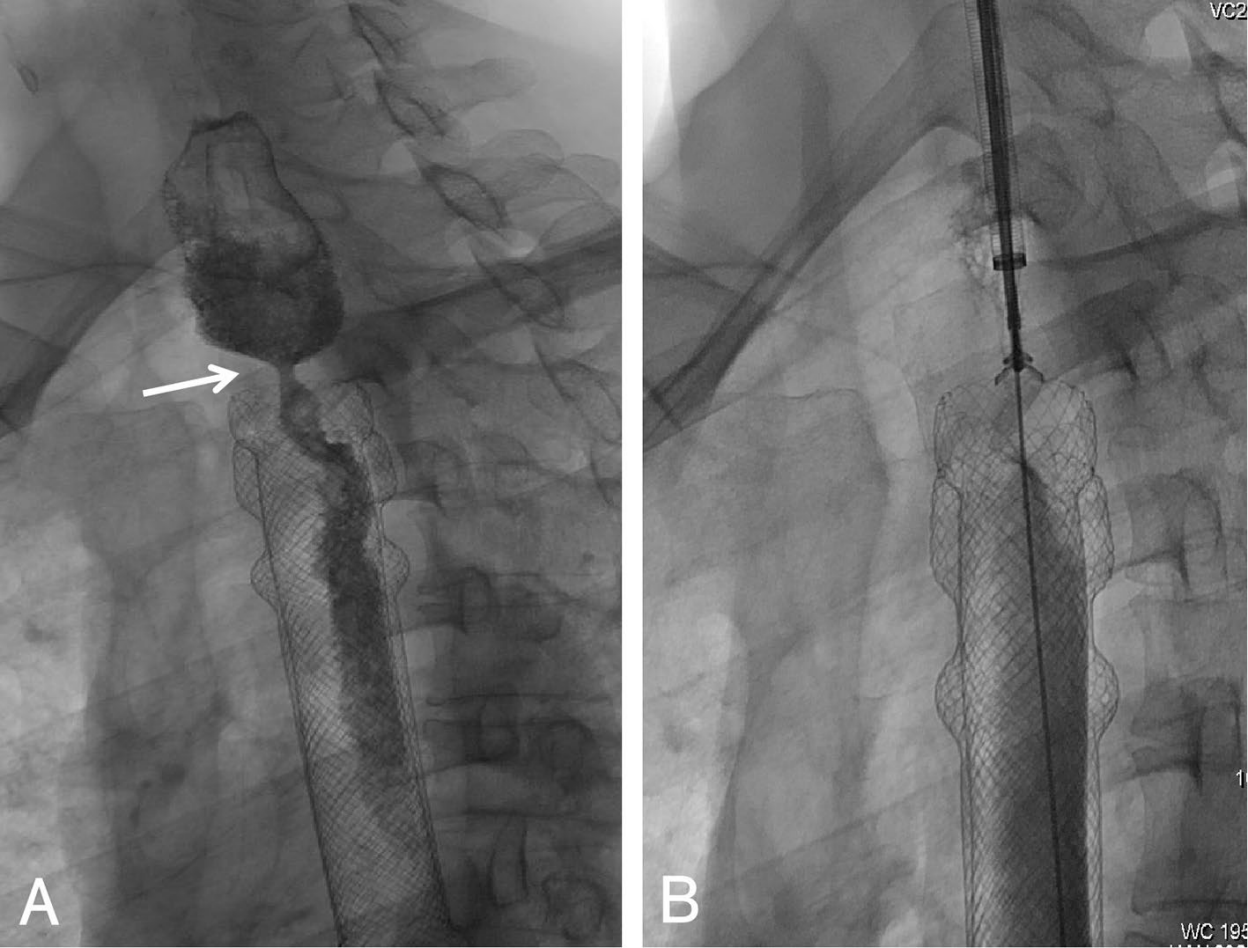
57-year-old male presented with level 3 dysphagia. (**A**) Catheter angiography demonstrated esophageal stenosis in the upper part of the esophageal stent. (**B**) The biopsy forceps was introduced through the sheath to clamp the samples, and granulation hyperplasia was confirmed by pathology.

For the procedure, the patients were supine on a digital subtraction angiography (DSA) examination bed. The patients’ oxygen inhalation was monitored and electrocardiograms were obtained. The esophageal tube was inserted under local anesthesia (oral) with 5 ml of 2% lidocaine. Using a 0.035-inch guidewire (Cook Bloomington IN, USA), the catheter was manipulated to the stomach through the esophageal stenosis; another angiography was performed through the catheter to measure the esophagus stricture or occlusion. The upper segment of the occluded part was dilated and the stricture length was recorded. Thereafter, the catheter was again manipulated toward the stomach and the guidewire was replaced with a super-stiff guidewire, along which a 10F long sheath (80 cm in length, Cook Bloomington IN, USA) was passed. The sheath end was pressed toward the narrow esophageal part. Then, the biopsy forceps was introduced into the sheath, opened, and pushed forward by 5–10 mm, after which the forceps was tightened to obtain the tissue sample (Figs. 1 and 2). After withdrawing the biopsy forceps, the diseased tissue was fixed in 10% formaldehyde and sent for histological examination. After 2–8 repetitions of theaforementioned process, at least two pieces of the tissue were successfully clamped. The specimens obtained were immediately evaluated by a pathologist, after which the operation was finished and the device was withdrawn. Then, the guidewire and catheter were manipulated with each other through nose-esophagus until they finally reached the stomach. The catheter was replaced with a nutritional tube, which was attached to the side of the face to hold it in place.

### Definition

The final results were obtained by reviewing the pathological results, final surgical operation findings, or follow-up medical images. The evaluation standards were as follows: (1) pathology results for the final surgical resection, (2) pathological results were accepted as corrected diagnosis such as malignant or specific benign results such granulation hyperplasia, hamartoma or tuberculosis; (3) for negative for malignancy, fibrous tissue, and inflammation results, follow-up imaging or endoscopy were used to decide the true or false results within 3 months.

### Statistical analysis

All data were recorded and analyzed using SPSS software (version 17.0, USA). Karnofsky scores were compared using a paired t-test, and significance was set at a *P*-value of 0.05.

### Ethics approval and consent to participate

This retrospective study was approved by the institutional review board of the First Affiliated Hospital of Zhengzhou University. All participants gave written informed consent that their data can be used for scientific purposes.

## Results

### General records

**Figure 3:**
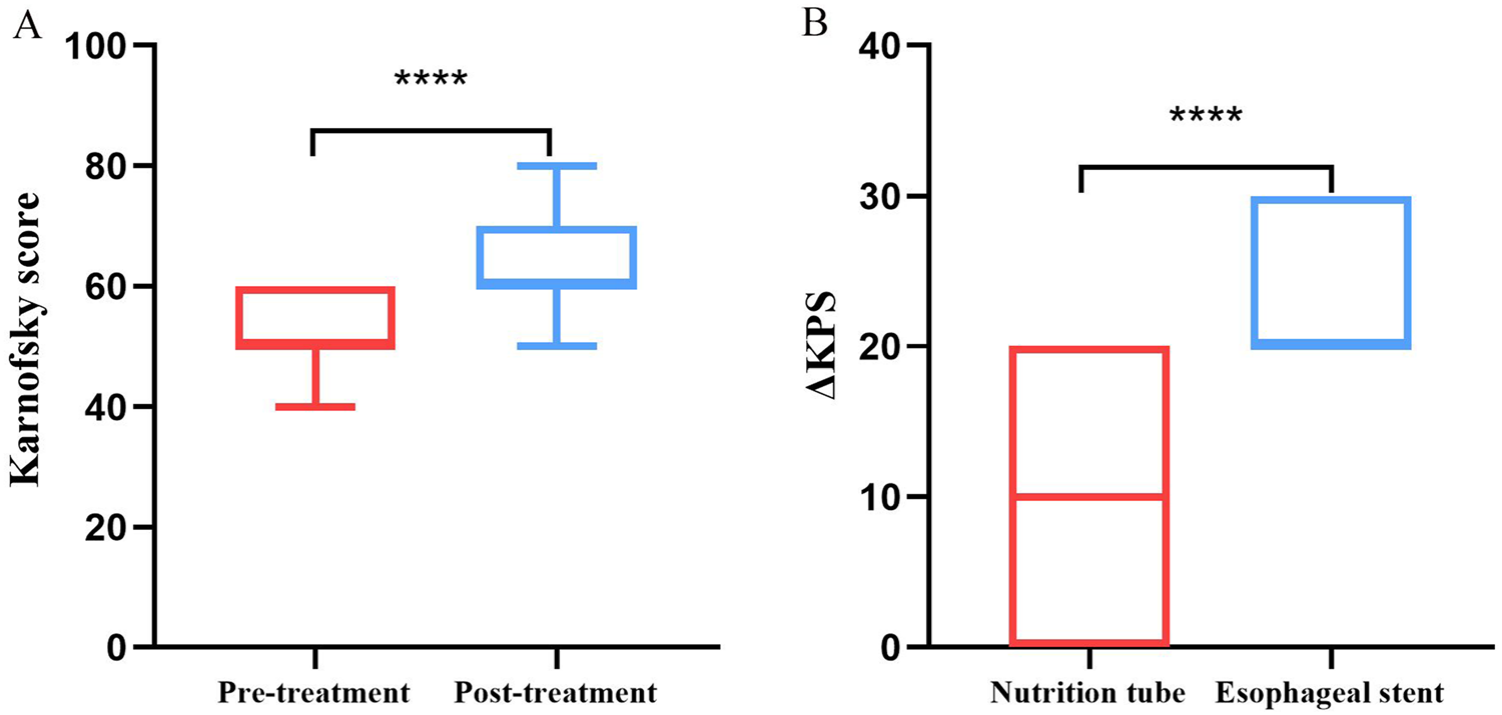
Comparison of preoperative and postoperative indicators. (**A**) The Karnofsky score was improved post-treatment, compared with pre-treatment (t = 11.22, *P* < 0.0001). (**B**) the ΔKPS was higher in 8 patients with esophageal stent implantation (21.3 ± 3.5), which was compared with the ΔKPS in 27 patients with nutritional tube implantation (9.3 ± 3.9, t = 7.87, *P* < 0.0001). ΔKPS = post-treatment Karnofsky score—preoperative Karnofsky score; ^*^P < 0.05, ^**^P < 0.01, ^***^P < 0.001, ^****^P < 0.0001.

The mean patient age was 74.3 years. The tumor was located in the upper, middle, and lower parts in 9, 19, and 7 cases, respectively. Stenosis length and tumor diameter were 48.7 mm (range: 21–80 mm) and 22.3 mm (range: 13–37 mm), respectively. The technical success of TTFB was 100%. The mean procedure duration was 23.2 min, resulting in a mean exposure dose of 7.2 mSv. The nutrition tube was used in 27 cases (77.1%), and an esophageal stent was inserted after TTFB in eight cases (22.9%). The pre- and post-procedure Karnofsky scores were 51.1 ± 7.6 (40–60) and 63.1 ± 9.0 (40–80), respectively, with there being a significant difference between the scores (t = 11.22, *P* < 0.0001). More detailed information is provided in Table 1 and Fig. 3. The subgroup showed that the Karnofsky of 8 patients with esophageal stent implantation increased by (21.3 ± 3.5), while the Karnofsky of 27 patients with nutrition tube placement increased by (9.3 ± 3.9). There was a statistical difference between the two groups (t = 7.87, *P* < 0.0001).

### Pathological results and complications

The mean number of biopsies was 5.2, and the number of samples from all patients was sufficient for diagnosis under the pathologist’s supervision. Diagnoses were missed in four patients (11.4%), of whom three showed epithelial dysplasia while the fourth patient showed a large amount of fibrous connective tissue that was suspected to be part of heterogeneous cells. Three of these patients received nasal feeding, and their physical status improved 3 weeks later, and the surgeries in these patients indicated constrictive esophageal cancer. The fourth patient was followed up and showed aggravated hyperplasia at the ends of the stent. After an improvement in the patient’s general health within 1 month, biopsy under gastroscopy was repeated, and it indicated squamous cell carcinoma. Thus, the sensitivity, specificity, and accuracy rate were 92.3% (24/26), 77.8% (7/9), and 88.6% (31/35). Complications occurred in 2 (5.7%) cases, with both patients showing some hematemesis (< 5 mL), and all patients' symptoms disappeared after injecting epinephrine (1 mg) through the sheath without further intervention. No esophageal perforation or massive hemorrhage was found.

## Discussion

Digestive endoscopy has significantly increased the rates of early detection of esophageal cancer or precancerous mucosal lesions^5^. Gastroscopy can allow direct observation of the lesions of the esophageal mucosa, especially for swelling and ulcerative lesions. EB can be performed simultaneously, which is generally accepted as one of the best ways to obtain the sample from esophageal cancer pretreatment, and the accuracy of endoscopic forceps biopsy was 60–95% in previous literature^6–8^. However, some patients cannot undergo EB due to anesthesia-related risks, age-related factors, severe occlusion, refusal, and other reasons, and appropriate diagnosis in these patients remains a clinical difficult problem. The most ideal condition in forceps biopsy is to obtain the least amount of biopsy tissue from the most typical part to reflect the overall nature of the lesion^9^. For patients with severe esophageal stenosis, the tumor information is hidden across the stenosis area and the approach used for obtaining pathological tissue out of the stenosis area is very important. In the past, Li TF et al. have reported that TTFB can be used to diagnose malignant obstructive jaundice; thus, it can hypothetically be used to diagnose esophageal strictures^10^. In addition, some patients don't even have water for several weeks, the general condition is very poor, nutritional support is still the most basic and important treatment for these patients. We can complete forceps biopsy and provide a nutrition tube or esophageal stent placement simultaneously under local anesthesia to improve the efficiency of diagnosis and treatment.

In this pilot study, 35 patients underwent TTFB for esophageal stenosis. The technical success rate was 100% and sufficient histologic specimens were obtained. The final pathological accuracy rate was 88.6%, which was satisfactory, and was within the accuracy range of previous reports^6–8^. The stenoses were relatively narrow, and biopsy forceps were manipulated closely to the beginning of the lesion at the head of the sheath so as to obtain samples out of lesion sidewalls. In terms of complications, two cases showed minor self-limiting hemorrhage, which is reasonable in comparison with that associated with the use of clamp forceps under endoscopy, and the safety was satisfactory^11^. However, TTFB has certain disadvantages such as unclear visualization of the mucosal surface, outline, and scope of the lesion and the incompatibility with narrow-band imaging technology. In addition, it is difficult to sample deeper submucosal lesions with such forceps. Diagnoses were missed in four patients, who were finally confirmed as showing cancer. This is still a practical problem, and a non-specific outcome in this examination cannot rule out the presence of malignant tumors, necessitating further evaluations. The location, method, quantity, forceps times, and specimen preparation may affect the assessment^12^. As for irradiation dose, 7.2 mSv is similar to the radiation dose in a standard CT chest scan (7 mSv)^13^, which is reasonable in our opinion, although the irradiation dose can be further reduced by using a small field of view and improving work efficiency.

In order to obtain more samples in patients with esophageal stenosis, our technical experience is as follows: (1) The long sheath must be introduced along a super-stiff guidewire and should be as close to the stenosis as possible. (2) After introducing the forceps through the outer sheath, the forceps can be opened and then pushed forward 5–10 mm into the stenosis so as to obtain more samples. (3) If the stenosis is very severe, forceps biopsy should be performed after dilatation with a small balloon dilator (diameter, 6–8 mm). Larger balloons (diameter ≥ 10 mm) are not recommended. When no stenosis indicates no support platform at the head of the sheath, it is easy to slide to the distal end of the stenosis. In such cases, the opening biopsy forceps cannot touch the sidewall of the lesion, and the forceps cannot get close to the diseased wall leading to sampling failure, or a large balloon can compress the esophageal cells leading to denaturation or necrosis. (4) Multiple biopsies may be required because the obstructive disease is actually caused by the lesion itself and the inflammatory edema around it. The biopsy forceps must be used in a very limited part of the middle of the lesion while obtaining the lesion tissue; otherwise, it may also collect inflammatory tissue. Therefore, it is necessary to forceps the stenosis in different parts and directions, but multiple biopsies do not imply the use of a higher number of clamps, which is also important to reduce complications while diagnosing diseases. (5) The diagnosis of tissue sections should be performed promptly by the pathology department, and the tissue dehydration time should not be too long; otherwise, the specimen will undergo compression, degeneration, and necrosis. The key of this technology is to pass the stenosis with guidewire, so it will be a huge challenge for patients with complete esophageal obstruction. Our experiences were: (1) The type of catheter tip was important. The end of 5F vertebral artery catheter is hard and flexible, which is equivalent to the adjustable mirror of the endoscopy; (2) All patients were fasting before operation, so that the mucosal edema would be relatively mild. In addition, dexamethasone can be used 3 days before operation; (3) Under fluoroscopy, filling defect and rat tail sign can be seen, therefore, the catheter can be skillfully rotated toward the narrowest part. Finally, the significance of enhanced CT should be emphasized, if the tumor invades the aortic arch and is accompanied by local necrosis, clamp biopsy should be extremely careful to avoid aortic rupture.

However, this study had its own limitations, such as the small number of patients, biased selection, and the absence of a prospective design and a control group.

In conclusion, TTFB is a safe and effective method for patients with severe esophageal obstruction under fluoroscopy, especially those who cannot undergo EB due to anesthesia-related risks, age-related factors, severe occlusion, or refusal to undergo endoscopy.

## Data Availability

The datasets used and/or analyzed during the current study are available from the corresponding author on reasonable request.

## Abbreviations

TTFB: Trans-oral trans-sheath forceps biopsy
EB: Endoscopic biopsy
